# *PLOS Medicine* 2018 Reviewer and Editorial Board Thank You

**DOI:** 10.1371/journal.pmed.1002765

**Published:** 2019-02-27

## Abstract

PLOS and the *PLOS Medicine* team would like to express our appreciation to the Editorial Board members, guest editors, and reviewers who participated in the manuscript assessment process for the journal in 2018.

PLOS and the *PLOS Medicine* team would like to sincerely thank all of our Editorial Board members, guest editors, and reviewers who participated in the manuscript assessment process for the journal in 2018. Your contributions of time and expertise support your research community, advance scientific progress, and continue to make *PLOS Medicine* a leader in its field. This past year, *PLOS Medicine* received the assistance of 75 Editorial Board members, 39 guest editors, and 731 reviewers in evaluating 2,620 manuscripts, which enabled the publication of 170 research articles and 57 magazine pieces (Fig 1).

**Fig 1:**
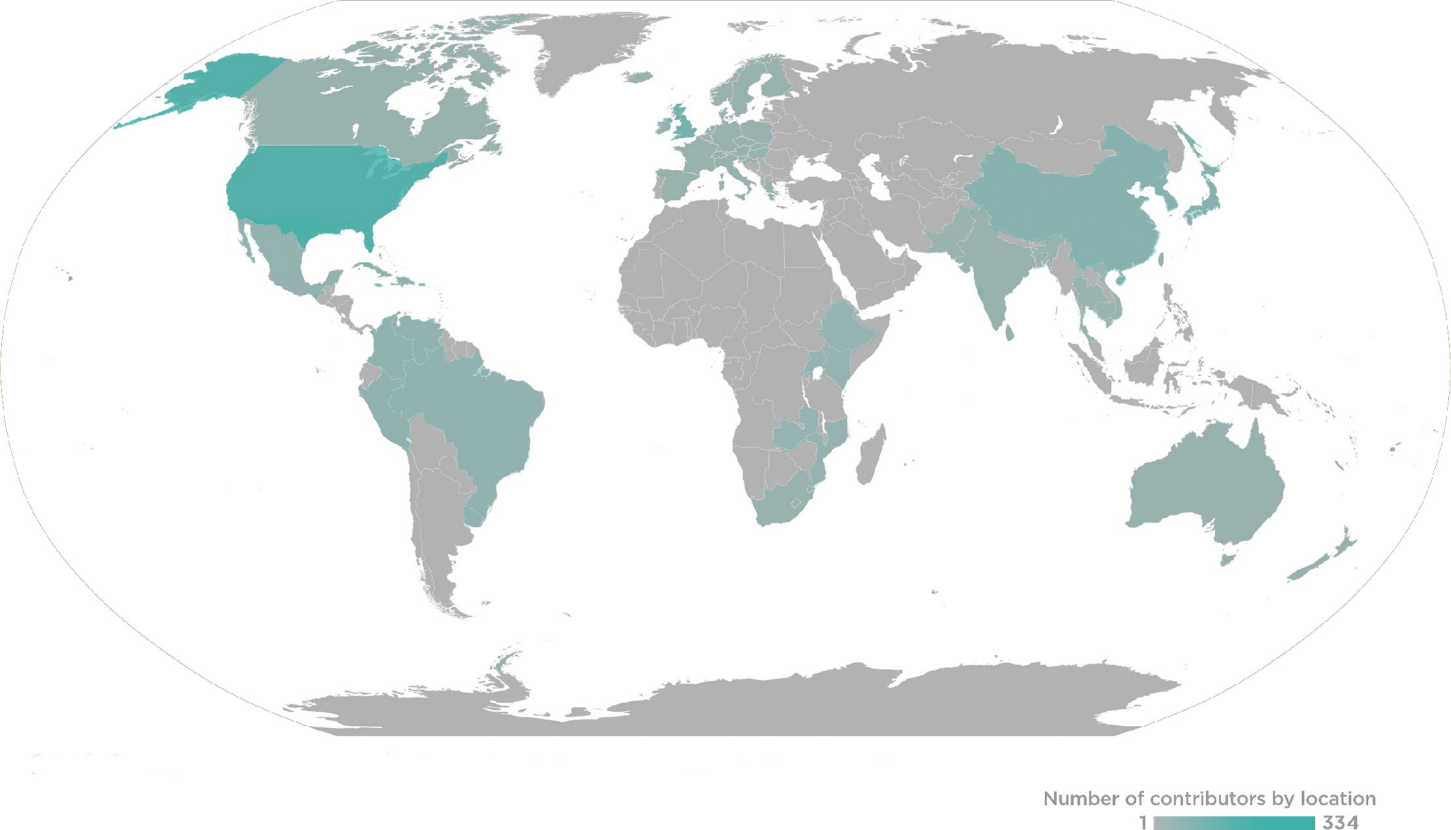
2018 *PLOS Medicine* Global Editor and Reviewer Locations.

We’re deeply grateful to everyone whose dedicated efforts support *PLOS Medicine* and Open Science. Thank you all for your work.
